# MTOR signaling and ubiquitin-proteosome gene expression in the preservation of fat free mass following high protein, calorie restricted weight loss

**DOI:** 10.1186/1743-7075-9-83

**Published:** 2012-09-14

**Authors:** Cassandra M McIver, Thomas P Wycherley, Peter M Clifton

**Affiliations:** 1 Commonwealth Scientific and Industrial Research Organisation (CSIRO), Food and Nutritional Sciences, PO Box 10041, Adelaide, Australia; 2 Sansom Institute for Health Research, University of South Australia, Adelaide, Australia; 3 Baker IDI, South Australia, Australia

**Keywords:** High protein diet, Fat free mass, Caloric restriction, Skeletal muscle, MTORC1, MuRF-1

## Abstract

Caloric restriction is one of the most efficient ways to promote weight loss and is known to activate protective metabolic pathways. Frequently reported with weight loss is the undesirable consequence of fat free (lean muscle) mass loss. Weight loss diets with increased dietary protein intake are popular and may provide additional benefits through preservation of fat free mass compared to a standard protein, high carbohydrate diet. However, the precise mechanism by which a high protein diet may mitigate dietary weight loss induced reductions in fat free mass has not been fully elucidated. Maintenance of fat free mass is dependent upon nutrient stimulation of protein synthesis via the mTOR complex, although during caloric restriction a decrease (atrophy) in skeletal muscle may be driven by a homeostatic shift favouring protein catabolism. This review evaluates the relationship between the macronutrient composition of calorie restricted diets and weight loss using metabolic indicators. Specifically we evaluate the effect of increased dietary protein intake and caloric restricted diets on gene expression in skeletal muscle, particularly focusing on biosynthesis, degradation and the expression of genes in the ubiquitin-proteosome (UPP) and mTOR signaling pathways, including MuRF-1, MAFbx/atrogin-1, mTORC1, and S6K1.

## Introduction

Current primary treatment strategies for obesity (BMI ≥30 kg/m2) are to consume a low-fat (<30% of total energy) diet with reduced caloric intake and participate in increased physical activity to create a negative energy balance. In overweight and obese individuals, even a modest reduction in weight (5 kg) can have significant health benefits including improved insulin sensitivity [1] pancreatic islet β-cell function [2], glucose homeostasis, blood pressure [3] and markers of cellular oxidative damage [4]. However, during lifestyle modification induced weight loss the loss of metabolically active fat free mass (FFM) is frequently reported as an undesirable consequence [5]. Emerging evidence suggests a high ratio of protein to carbohydrate in a low-fat, caloric restricted (CR) diet may mitigate FFM reductions during weight loss through increasing muscle protein synthesis and/or reducing protein catabolism, thereby improving net muscle protein balance [6]. The precise mechanism by which an increased dietary protein intake may mitigate weight loss induced reductions in FFM has not been fully elucidated.

During eucaloric conditions the continual breakdown of protein that occurs in the body’s organs and vital tissues is replenished in the post-absorptive state via supply of amino acids derived primarily from the skeletal muscle component of FFM [7]. In turn, during fed states, skeletal muscle proteins are replenished through a feeding stimulated increase in muscle protein synthesis that occurs almost exclusively due to the protein constituent of the ingested food [8]. Once requirements for adequate substrate to replenish skeletal muscle are exceeded, the feeding induced muscle protein response is inhibited [9]. Providing there is adequate dietary protein intake, muscle protein gains which occur in the fed state balances the loss of muscle protein which occurs in the post absorptive state allowing day to day skeletal muscle mass to remain relatively constant [7,10]. However, excess nutrient intake of amino acids and glucose, beyond the bodies requirement to maintain homeostasis and energy production for cellular processes, leads to insulin resistance in skeletal muscle via a dysregulation of the insulin signaling pathway and potentially promoting protein catabolism [11,12].

The FFM reduction that typically occurs during CR weight loss also implies a negative net skeletal muscle protein balance. There are a number of plausible mechanisms, several of them mediated by dietary protein that may provide some explanation for the negative net protein balance. These include an elevated rate of breakdown of muscle protein in response to caloric restriction via up-regulation of protein catabolism enzymes [13,14]; an inadequate per-meal dose of dietary protein and subsequently reduced maximal post-meal rate of muscle protein synthesis [8,15]; a reduced number of meals/protein ingestions throughout the day and subsequently a reduced number of periods of elevated muscle protein synthesis [16,17]; and/or a reduced rate of post-meal muscle protein synthesis relating to the type/quality of dietary protein being ingested [18,19]. Although it is likely an increased dietary protein intake during CR mitigates reductions in FFM through one or more of these mechanisms, further well controlled randomised clinical trials are required to investigate the contribution of each of these factors and whether an optimal dietary configuration exists that can completely stave off FFM loss. This review evaluates current evidence suggesting an increased dietary protein intake during CR weight loss may mitigate FFM reduction in overweight and obese persons via decreased protein catabolism and improve metabolic factors when compared with standard protein, high carbohydrate, CR weight loss diets. Candidate mechanisms are discussed with a focus on the ubiquitin-proteosome (UPP) and the mammalian target of rapamycin (mTOR) signaling pathways and their association with CR and retention of FFM.

### High protein, caloric restriction and weight loss in humans

A high protein, CR diet is typically considered to constitute ~30% daily total energy from protein, 40% from carbohydrate and 30% from fat, with caloric intakes ~6000 kj/day (1400 kcal) for women and 7000 kj/day (1600 kcal) for men. A standard protein or high carbohydrate diet is typically comprised of ~15% daily total energy from protein, 55% from carbohydrate and 30% from fat.

A high protein, low fat diet compared to a high carbohydrate, standard protein CR diet has been demonstrated to result in greater weight loss [20-25] and metabolic advantages (greater reductions in total cholesterol and triglycerides in men [26,27] and reduced FFM loss in women [28-31]) (Table 1). However, there are also a number of studies that have shown no differences in total weight loss [28,30,32-34] or retention of FFM [22,23,35-37] when high protein CR diet regimes are compared to a high carbohydrate diet. Further confounding evidence also exists with at least one study showing a greater loss of FFM in hyperinsulinemic males following a high protein diet compared the standard-protein diet [38]. However, this loss was only 0.9 kg and the authors conclude that hyperinsulinemic obese subjects, in contrast to normoinsulinemic subjects, seem to achieve better weight reduction, less decline in energy expenditure, and normalization of insulin levels on a high protein compared to a isocaloric high carbohydrate diet. 

**Table 1:** **Change (Δ) in fat free mass (FFM), fat mass and total body weight ± standard error of the mean in women from previous studies examining the effects of increased dietary protein intake and weight loss on body composition (Farnsworth *et al.* [ **[28]**]; Luscombe-Marsh *et al.* [ **[65]**]; Noakes *et al.* [ **[25]]; **Layman *et al.* [ **[29]**]; Piatti *et al*., 1994 [ **[31]])

**Author**	**Population**	**N (M/F)**	**Intervention**	**ΔFFM†**	**ΔFat mass†**	**ΔTotal body weight†**
Farnsworth *et al.*[28]	Healthy Obese	57 (14/43)	HP Diet vs SP Diet (equal% energy from fat) 12 weeks energy restriction	HP Diet −0.1 ± 0.3**	HP Diet −6.6 ± 0.5	HP Diet −6.6 ± 0.5
			4 weeks energy balance	SP Diet −1.5 ± 0.3	SP Diet −7.1 ± 2.0	SP Diet −7.4 ± 0.5
Luscombe-Marsh *et al.*[65]	Healthy Obese	57 (25/32)	LF-HP Diet vs HF-SP Diet 12 weeks energy restriction	LF-HP Diet −2.2 ± 0.5*	LF/HP Diet −4.3 ± 0.8	LF-HP Diet −7.8 ± 0.8
			4 weeks energy balance	HF-SP Diet −3.1 ± 0.5	HF/SP Diet −4.8 ± 1.2	HF-SP Diet −7.9 ± 1.3
Layman *et al.*[29]	Healthy Obese	25 F	High protein vs High Carbohydrate (similar% energy from fat)	HP Diet −0.88 ± 0.3**	HP Diet-5.6 ± 0.5	HP Diet −7.53 ± 1.4
			10 weeks energy restriction	HC Diet −1.21 ± 0.6	HC Diet-4.7 ± 0.7	HC Diet −6.96 ± 1.36
Noakes *et al.*[25]	Healthy Obese	100 F	HP vs SP Diet (equal% energy from fat)	HP Diet −1.5 ± 0.3	HP Diet −5.7 ± 0.6	HP Diet −7.6 ± 0.4
			12 weeks energy restriction	SP Diet −1.8 ± 0.3	SP Diet −4.5 ± 0.5	SP Diet −6.9 ± 0.5
Piatti *et al.*[31]	Healthy Obese	25 F	HP vs SP Diet (equal% energy from fat)	HP Diet −1.40 ± 0.6**	HP Diet −3.2 ± 0.6	HP Diet −4.5 ± 0.4
			21 days energy restriction	SP Diet −3.02 ± 0.6	SP Diet −3.3 ± 0.5	SP Diet −6.4 ± 0.9

†Data from women only.

**Indicates a significant retention of lean mass in the HP diet group and *indicates a trend.

An increased thermogenic effect may give high protein diets a metabolic advantage over high carbohydrate diets. Dietary protein has been shown to have a substantially greater effect on basal thermogenesis (compared to carbohydrate or fat) [39,40] and nitrogen turnover is increased (indicating protein synthesis is elevated) [39]. In eucaloric studies a short term (3-month) increase in daily protein intake significantly decreased body fat and preserved lean mass in healthy lean participants [41]. However, long term high protein dietary intake (>1.8 g.kg^-1^ day^-1^) in newly diagnosed insulin dependent diabetes mellitus (DM) and healthy lean participants was found to increase plasma insulin concentrations and decrease glucose oxidation resulting in a state of insulin resistance and glucose intolerance but these were small observational studies and not controlled interventions [42,43].

### Caloric restriction and lean muscle in humans

Although studies unanimously observe favourable benefits from CR for reducing body weight and fat mass, a confounding complication is the reduction of FFM (muscle atrophy) which is frequently reported [5,44]. FFM is the main determinant of resting metabolic rate (RMR) [45], which suggests a decrease in FFM could hinder the progress of weight loss and may predispose to weight regain [46,47]. Furthermore, lost FFM is typically not fully recovered in individuals who regain weight, predisposing them to the burden of “sarcopenic obesity” [48]. In women, deterioration in muscle performance has been observed as early as perimenopause, increasing their vulnerability to sarcopenia compared to age-matched men [49]. Loss of FFM may also have detrimental effects in older persons whereby accelerated muscle loss correlates negatively with functional capacity for independent living [50]. Skeletal muscle atrophy, caused by an imbalance of protein synthesis and catabolism, is readily apparent in conditions such as uncontrolled diabetes, cancer cachexia, spinal cord injury and muscular disuse. A review of dietary protein for muscle atrophy in cachexia by Op den Kamp *et al.*[51] found that supplementation with dietary protein (>1.5 g.kg^-1^ day^-1^) alone or in combination with exercise training maintains or even improves muscle mass in these patients. In addition, protein supplementation (30 g/d) during weight maintenance has been demonstrated to limit weight regain following weight loss [52].

**Figure 1:**
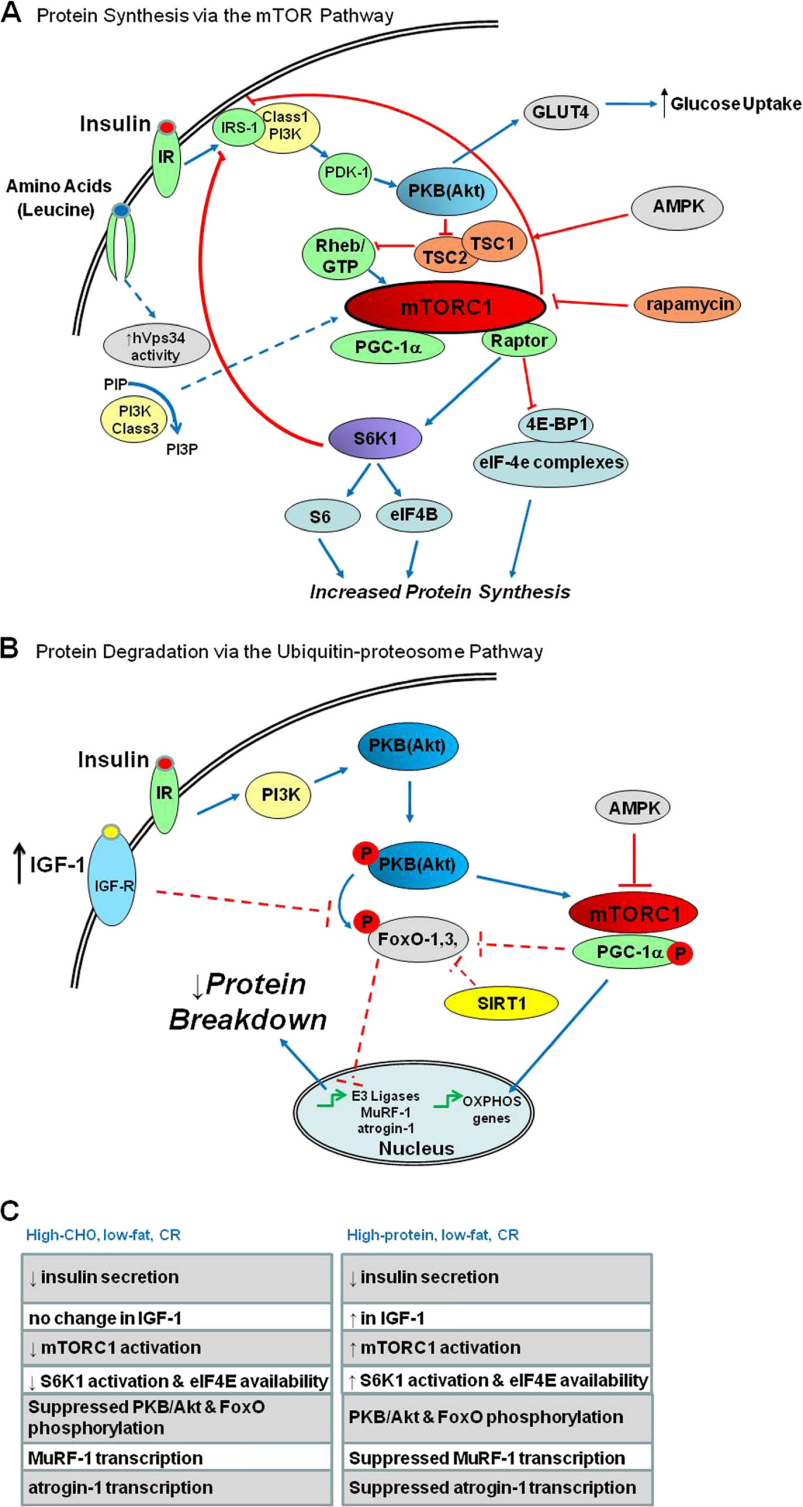
** A schematic representation depicting A; the protein synthesis pathway in skeletal muscle involving the mammalian target of rapamycin complex 1 (mTORC1).** Insulin, and amino acids (including leucine) initiate activation of a cascade of protein and lipid kinases ultimately resulting in enhanced mTOR activity, facilitating the phosphorylation of S6K1 and hyper-phosphorylation of 4E-BP, resulting in enhanced availability of eIF4E for binding eIF4G and forming an active eIF4F complex resulting in increased protein synthesis [adapted from Layman [88], Anthony *et al.*[89], Drummond *et al.*[61], Um *et al.*[98] and Kimball [90,93] and **B**; our proposed mechanism whereby high protein calorie restricted weight loss increases IGF-1 activating the PI3K/Akt pathway, thereby phosphorylating (P) FoxO transcription factors and down-regulating the expression of E3 enzymes atrogin-1 and MuRF-1, leading to a reduction in protein degradation in skeletal muscle cells. PGC-1α, SIRT1 and AMPK are also proposed to inhibit the expression of FoxO transcription factors and therefore suppress protein breakdown [adapted from Lecker *et al.*[70], Bodine *et al.*[99], Anthony *et al.*[89] and Blagosklonny *et al.*[62]. Dashed lines indicate an interaction with an unknown mechanism. Red lines indicate an inhibitory signal to the pathway, and **C**; Summarisation of protein biosynthetic and degradation events following standard protein, high carbohydrate compared to high protein, calorie restricted weight loss.

The mechanisms by which an increased dietary protein intake mitigates dietary weight loss induced reductions in FFM, as found in some studies, may be explained by examination of the molecular pathways involved in the control of muscle protein synthesis (hypertrophy) and breakdown (atrophy). Insulin-like growth factor 1 (IGF-1) and PKB/Akt are believed to play key roles as central targets in the protein synthesis [53] and degradation pathways [54]. Amino acids and insulin activate muscle protein synthesis via a complex serine-threonine protein kinase, mammalian target of rapamycin (mTOR) signaling pathway (Figure 1A) resulting in cellular mass growth [55]. Nutrient overload, in particular increased fat and elevated circulating amino acids, have been shown to cause β-cell compensation and increased activation of mTOR which can lead to insulin resistance in peripheral insulin-responsive tissues [56]. Beyond dysregulation of glucose homeostasis, impaired insulin signaling in muscle contributes to the muscle loss observed in obesity by promoting protein catabolism through the expression of ubiquitin ligases and hence a possible explanation as to why high mTOR activity in muscles of obese humans and mice does not result in muscle hypertrophy (Reviewed in [11]). Individuals with type 2 DM may also have impaired insulin-mediated protein synthesis [57,58] as amino acid signaling to mTOR complex 1 (mTORC1) requires co-stimulation with insulin [59] which generates an inhibitory feedback loop on insulin receptor substrate proteins [56]. This is in contrast to older adults (>65 years) whereby a blunted muscle protein synthesis response has been observed compared with young adults (<30 years) following resistance exercise [60], indicating that older adults may have an impaired ability to respond to a protein anabolic stimulus resulting in acute dysregulation of this signaling pathway [61]. A lack of nutrients (i.e. fasting and possibly CR) have also been proposed to activate adenosine monophosphate (AMP)-activated kinase (AMPK) and nicotinamide adenine dinucleotide (NAD^+^)-dependent deacetylases, such as (Sirtuin 1) SIRT1, which in turn suppresses the mTOR pathway [62]. 

There is a strong indication that dysregulation of mTOR signaling, and therefore a reduced ability to maintain protein synthesis, occurs in translation initiation, as older subjects have lower p70 ribosomal S6 kinase 1 (p70S6K1) phosphorylation and blunted extracellular signal-regulated kinase 1 and 2 (ERK1/2) and mitogen-activated protein (MAP) kinase-interacting kinase 1 (MNK1) signaling compared to younger subjects following exercise at the same relative intensity [63]. Nutrient excess, in particular high-fat diets, can reduce the ability of leptin and insulin to promote mTORC1 activity and reduce food intake [11] indicating that diet quality may be a driving factor in our ability to maintain protein synthesis rather than age. Others have found no alteration of protein levels of IRS-1, mTOR or p70S6K in obese and type 2 DM skeletal muscle compared to age-matched lean participants, although a reduction in mTOR phosphorylation in obese and type 2 DM groups and reduced System L transporters, amino acid transporter/solute carrier family 43, member 2 (LAT4) and solute carrier family 3-activator of dibasic and neutral amino acid transport, member 2 (CD98hc) in the type 2 DM group have been reported [64].

Numerous studies have shown that women tend to lose less FFM (e.g. 0.1 kg compared to 1.5 kg) with high protein CR diets than with a standard protein diet (Table 1) [28,29,31,65], although others show no significant differences in total weight loss or total fat loss between groups. The FFM sparing ability from increased protein:carbohydrate ratio in a CR diet may be mediated by the effect of protein intake on insulin secretion [66] and proteolysis [13]. Proteolysis mainly occurs via the ubiquitin- proteosome pathway (UPP), which degrades both cytosolic and nuclear proteins [67], as well as myofibrillar proteins [68], which comprise most of the protein in adult skeletal muscle [69]. Studies have shown during fasting and possibly other insulin-deficient states, a reduction in protein synthesis and increased proteolysis occur through decreased signaling by the PI3K/Akt pathway [70] as IGF-1/insulin blocks transcriptional up-regulation of key mediators of skeletal muscle atrophy [54]. IGF-1/insulin also inhibits the expression of two E3 ligases, muscle atrophy F-box protein (MAFbx/atrogin-1) and muscle-specific RING finger protein 1 (MuRF-1) [71].

### Ubiquitin-proteosome pathway, CR and muscle protein degradation

During CR a decrease (atrophy) in skeletal muscle may be driven by a homeostatic shift favouring protein catabolism which may have a significant impact on FFM retention. Muscle protein degradation is a complex process in which lysosomal proteases, the Ca^2+^ − dependent proteases, the caspases and the UPP have been implicated [72]. Autophagic and proteosomal activity decline during aging and may contribute to age-related muscle loss [73,74]. In contrast, evidence in rodents suggests that CR increases the activity and effectiveness of these cellular quality control processes through prevention of an increase in protein carbonyl accumulation [75], delaying the age associated increase of chymotrypsin-like activity, an indicator of proteaosome activity [76]. The stimulation of proteolysis observed during atrophy has been shown to be partly due to the activation of the UPP [77] and therefore this pathway may be pivotal in FFM loss during weight loss. In rodents, CR has been shown to decrease plasma insulin and serum IGF-1 concentrations up to 40% [78] (reviewed in [79]), which may impact negatively on skeletal muscle. As IGF-1 has been shown to block transcriptional up-regulation of a number of ubiquitin-ligases [54], a decrease in circulating IGF-1 would result in up-regulation of MAFbx/atrogin-1 and MuRF-1 in skeletal muscle leading to increased proteolysis and hence FFM loss. In humans, long term severe CR (1 and 6 years) did not reduce serum IGF-1 levels. However, a reduction in protein intake (1.67 to 0.95 g.kg^-1^.day^-1^) during CR for 3 weeks in a small number of volunteers resulted in a reduction in serum IGF-1 (152 ng.mL^-1^) [78]. A 12 week CR high protein, high red meat diet in men also found IGF-related peptides significantly increased total (HP 23%; HC 18%) and bioactive (HP 18%; HC 15%) IGF-1 [80] compared to a high carbohydrate (standard protein) diet. In weight stable older, postmenopausal women an increased dietary protein intake (30 g whey supplement/day for 2 years) without CR significantly increased serum IGF-1 compared to placebo [81] indicating that increased dietary protein, through its ability to increase IGF-1 during CR, may prevent increased proteolysis via inhibition of up-regulation of key ubiquitin-ligases (Figure 1B).

High protein, low-carbohydrate diets are also accompanied by increased stimulation of glucagon and insulin production within the endocrine pancreas, high glycogen turnover and to some extent stimulation of gluconeogenesis [42,43]. Undefined insulin levels may stimulate PI3K/Akt, phosphorylating the forkhead transcription factor (FoxO) resulting in cytoplasmic retention and the repression of target gene expression [82]. Therefore a high protein CR diet for weight loss may suppress key regulatory elements of the UPP. Initiation of FoxO1 phosphorylation by PKB in skeletal muscle may decrease FoxO’s ability to stimulate the expression of the ubiquitin ligase (E3) enzymes MAFbx/atrogin-1 and MuRF-1 [83] which, when up-regulated, are essential for protein degradation and hence muscle atrophy (Figure 1B). Following ultra-endurance exercise and exercise in combination with weight loss the amount of ubiquitin-conjugated proteins and chymotrypsin-like activity has been shown to be decreased [72,84]. Up-regulation of mRNA transcripts MuRF1, F-box and C2 proteosome subunits were also observed as were the autophagy regulatory proteins Atg7 and LC3B [84] indicating that during ultra-endurance exercise cellular quality control processes are required to possibly improve skeletal muscle function by repairing muscle damage. Therefore we propose that high protein, CR weight loss diets may phosphorylate PKB/Atk and FoxO leading to suppression of MAFbx/atrogin-1 and MuRF-1 transcription resulting in deceleration of proteolysis that occurs during CR in skeletal muscle (Figure 1C) resulting in a preservation of FFM.

### Dietary protein intake and protein synthesis

Recently it was shown that a short-term, isocaloric high protein diet (10 days, >130 g/day protein) increased whole body protein turnover and amino acid (leucine) oxidation with no increase in muscle protein synthesis or mitochondrial function in either young (<25 years) or older (>70 years) participants. This indicates that higher protein intakes may stimulate protein synthesis following meal ingestion but may not enhance basal protein synthesis. However, postabsorptive protein catabolism (both breakdown and amino acid oxidation) was increased during the high protein diet [85]. Little is known about whether this occurs with high protein diets during CR. Dairy products, which contain whey protein, are often a key component of a high protein, low-fat diet. Whey contains both angiotensin-converting enzyme (ACE) inhibitor activity and a high concentration of leucine, a branched-chain amino acid (BCAA) [86]. The inclusion of whey protein in CR regimes may result in greater preservation of skeletal muscle and accelerated loss of adipose tissue during negative energy balance [86,87]. There is also increasing evidence to suggest that BCAA’s, particularly leucine, have a significant role in metabolic regulation beyond their fundamental role as substrates for protein synthesis [86,88].

The regulation of skeletal muscle protein turnover also involves the interactions of gene transcription and translation and numerous pre- and post-transcriptional modifications [61]. Leucine stimulates signal transduction pathways that modulate mRNA translation initiation thereby up-regulating protein synthesis [89-91]. Alpha-ketoisocaproic acid, a leucine metabolite has been identified to stimulate the phosphorylated heat- and acid-stable protein (PHAS-I), a recently discovered regulator of translation initiation during cell mitogenesis [92]. The action of leucine in the insulin signaling pathway is initiated by mTORC1 [59,93], which is activated by a variety of hormones (e.g. insulin) and nutrients (e.g. amino acids) that stimulate cell growth and proliferation, while it is repressed by other hormones (e.g. glucocorticoids) [94]. The mTOR complex also controls important functions in peripheral organs including muscle oxidative metabolism, white adipose tissue differentiation, β-cell dependent insulin secretion [95] and muscle autophagy [11].

Increases in amino acid concentrations stimulate mTOR kinase activity (Figure 1A) to initiate phosphorylation of the inhibitory eukaryotic initiation factor 4E binding protein (4E-BP1) causing it to dissociate from the eukaryotic translational initiation factor E (eIF4E). Once dissociated, eIF4E is available to bind with eIF4G to form an active initiation complex. Leucine has been suggested to stimulate protein synthesis in skeletal muscle through both insulin-dependent and independent mechanisms. The insulin-dependent mechanism is associated with signaling through mTOR via phosphorylation of eIF4E-binding protein1 (4E-BP1) and S6K1 [59], in contrast to the insulin-independent effect by an unknown mechanism that may involve phosphorylation of eIF4G and/or its association with eIF4E [90]. However, Amino acid availability also increases intracellular Ca^2+^ levels which can activate mTORC1 by means of a Ca^2+^/calmodulin-mediated activation of a Class III PI 3-kinase, human vacuolar protein sorting 34 (hVps34) [96,97], phosphorylating both S6K1 and 4E-BP1. As part of this mTOR signaling cascade, IGF-1 has also been shown to activate translation and muscle protein synthesis via tuberous sclerosis 2/tuberin (TSC2). PKB works by phosphorylating TSC2 at phosphorylation sites that are distinct from AMPK phosphorylation sites [53]. This pathway has been suggested to be suppressed or deactivated by caloric restriction via the activation of AMPK and SIRT1, which also occurs with Metformin administration (mimics CR), and possibly deactivating the insulin/PI3K pathway [62]. To what extent obesity causes a dysregulation of this pathway is unclear. Less is also clear about how high protein CR weight loss impacts on this pathway.

## Conclusion

The mechanism behind the preservation of lean muscle following high protein, CR weight loss has been proposed to be the increased consumption of amino acids, in particular leucine, stimulating increased muscle protein synthesis via the mTOR signaling pathway. However, in contrast to findings in animal models, studies in humans have found no alteration of protein levels of IRS-1, mTOR or p70S6K in obese and type 2 DM skeletal muscle compared to age-matched lean participants and CR may in fact deactivate this pathway. Therefore stimulation of the mTOR pathway to increase protein synthesis does not fully explain the retention of FFM seen in high protein, weight loss dietary intervention studies. Here we propose that the key mechanism may involve the suppression of regulatory elements of the UPP in skeletal muscle to prevent atrophy. As a reduction of FFM appears to be a confounding complication to weight loss, understanding the underlying biological mechanisms that occur in response to macronutrient composition may help us to provide more comprehensive dietary information for health care providers and individuals to facilitate healthy weight loss and long term weight maintenance for the treatment of obesity.

## Abbreviations

4E-BP1: Eukaryotic initiation factor 4E binding protein; ACE: Angiotensin-converting enzyme; Akt: Serine/threonine protein kinase; AMP: Adenosine monophosphate; AMPK: AMP-activated kinase; Atg7: Autophagy-related protein 7; BCAA: Branched-chain amino acid; BMI: Body mass index; Ca^2+^: Calcium; CD-36: Cluster of differentiation, CD36 molecule (thrombospondin receptor); CD98hc: Solute carrier family 3 (activators of dibasic and neutral amino acid transport), member 2; CR: Caloric restriction; DM: Diabetes Mellitus; eIF4E/G: The eukaryotic translational initiation factor E and G; ERK1/2: Extracellular signal-regulated kinase 1 and 2; FFM: Fat free mass; FoxO1/3: Forkhead transcription factor 1 and 3; GLUT4: Glucose transporter 4; hVps34: Human vacuolar protein sorting 34; IGF-1: Insulin-like growth factor 1; IGF-R: Insulin-like growth factor 1 receptor; IR: Insulin receptor; IRS-1: Insulin receptor substrate 1; LAT4: System L amino acid transporter/solute carrier family 43, member 2; LC3B: Iutophagy-related ubiquitin-like modifier; MAFbx/atrogin-1: Iuscle atrophy F-box protein; MAP: Iitogen-activated protein; MNK1: MAP kinase-interacting kinase 1; mTOR: Mammalian target of rapamycin; mTORC1: MTOR complex 1; MuRF-1: Muscle ring finger-1; NAD^+^: Nicotinamide adenine dinucleotide; P70S6K1: P70 ribosomal S6 kinase 1; PDK-1: Pyruvate dehydrogenase kinase, isozyme 1; PGC-1α: Peroxisome proliferator-activated receptor γ co-activator 1 alpha; PHAS-1: Phosphorylated heat- and acid-stable protein; PI3K: Phosphatidylinositol 3-kinase/Akt; PKB: Protein kinase B; PYY3-36: Peptide YY 3–36; Raptor/RPTOR: Regulatory associated protein of MTOR, complex 1; Rheb/GTP: Ras homolog enriched in brain/guanosine 5'-triphosphate; RMR: Resting metabolic rate; S6K1: S6 kinase 1; SIRT1: The mammalian ortholog of Sir2, sirtuin 1; TSC1/2: Tuberous sclerosis 1/2; UCP3: Uncoupling protein 3; UPP: Ubiquitin-proteosome pathway.

## Competing interests

The authors declare that they have no competing.

## Authors’ contributions

CMM, TPW and PMC drafted, edited and approved the final manuscript.
